# Decoding Immune Signature to Detect the Risk for Early-Stage HCC Recurrence

**DOI:** 10.3390/cancers15102729

**Published:** 2023-05-12

**Authors:** Aswathy R. Devan, Bhagyalakshmi Nair, Manu Kanjoormana Aryan, Vijayastelar B. Liju, Joel Joy Koshy, Bijo Mathew, Arun Valsan, Hoon Kim, Lekshmi R. Nath

**Affiliations:** 1 Department of Pharmacognosy, Amrita School of Pharmacy, Amrita Vishwa Vidyapeetham, AIMS Health Science Campus, Kochi 682041, Kerala, India; aswathyrd@aims.amrita.edu (A.R.D.); bhagyanair34@gmail.com (B.N.); joelk0946@gmail.com (J.J.K.); 2 Department of Immunology, Amala Cancer Research Centre, Thrissur 680555, Kerala, India; manukanjoor@gmail.com; 3 The Shraga Segal Department of Microbiology-Immunology and Genetics, Faculty of Health Sciences, Ben Gurion University of the Negev, Beer Sheva 84105, Israel; lijubsteltar@gmail.com; 4 Department of Pharmaceutical Chemistry, Amrita School of Pharmacy, Amrita Vishwa Vidyapeetham, AIMS Health Science Campus, Kochi 682041, Kerala, India; bijovilaventgu@gmail.com; 5 Department of Gastroenterology and Epatology, Amrita Institute of Medical Science, Kochi 682041, Kerala, India; arunvalsan@aims.amrita.edu; 6 Department of Pharmacy, and Research Institute of Life Pharmaceutical Sciences, Sunchon National University, Suncheon 57922, Republic of Korea

**Keywords:** HCC, curative treatment, recurrence, immunosuppression, adjunct immunotherapy, immune checkpoint inhibitors

## Abstract

**Simple Summary:**

Locoregional therapies such as surgical resection, liver transplantation, and ablative techniques are preferred for early-stage HCC management. However, 70–80% of patients report HCC recurrence within 5 years of curative treatment. During the initiation and establishment of carcinogenesis, malignant cells suppress the anti-tumor immune surveillance system of the host. Therefore, modification of the immune contexture plays a crucial role in prognosis and recurrence. Following curative treatment, enhancement of effector immune cells confers effective anti-tumor immunity and good prognosis, while suppression of effector immune cells and enrichment of immunosuppressant cells promotes poor prognosis and recurrence. A combination of immunotherapeutic approaches with curative or systemic treatment may enrich effector immune molecules, surpass the immunosuppressive signals, and improve the treatment response.

**Abstract:**

Hepatocellular carcinoma (HCC) is often recognized as an inflammation-linked cancer, which possesses an immunosuppressive tumor microenvironment. Curative treatments such as surgical resection, liver transplantation, and percutaneous ablation are mainly applicable in the early stage and demonstrate significant improvement of survival rate in most patients. However, 70–80% of patients report HCC recurrence within 5 years of curative treatment, representing an important clinical issue. However, there is no effective recurrence marker after surgical and locoregional therapies, thus, tumor size, number, and histological features such as cancer cell differentiation are often considered as risk factors for HCC recurrence. Host immunity plays a critical role in regulating carcinogenesis, and the immune microenvironment characterized by its composition, functional status, and density undergoes significant alterations in each stage of cancer progression. Recent studies reported that analysis of immune contexture could yield valuable information regarding the treatment response, prognosis and recurrence. This review emphasizes the prognostic value of tumors associated with immune factors in HCC recurrence after curative treatment. In particular, we review the immune landscape and immunological factors contributing to early-stage HCC recurrence, and discuss the immunotherapeutic interventions to prevent tumor recurrence following curative treatments.

## 1. Introduction

Despite significant progress in diagnostic and therapeutic interventions, hepatocellular carcinoma (HCC) remains as the deadliest malignancy with the highest recurrence rate [1]. Inflammation is a hallmark of HCC, closely linked with all stages of HCC development starting from fatty liver, steatohepatitis, to hepatocarcinogenesis. Chronic, unresolved inflammation provides an immunosuppressive tumor microenvironment, which further promotes tumorigenesis and metastasis [2,3]. As a cell intrinsic genetic disease, most of the curative and systemic therapies focus directly on the tumor cells. However, the unnoticed inflammatory tumor microenvironment plays a crucial role in determining the therapeutic response and survival benefit in patients [4,5].

At the early stage of Barcelona Clinic liver cancer (BCLC), stage 0/stage A, locoregional therapies such as surgical resection, liver transplantation, and ablative techniques are preferred. These offer an improved five year survival rate of 70–80% [6]. Within two years of follow-up, 30–50% of patients report recurrence and 50–70% develop recurrence within five years of the treatment [7,8]. Conventionally, the anatomic extent of the tumor burden is considered to characterize prognosis and predict recurrence. In addition to the oncological factors, immunity plays a leading role in controlling the malignant transformation of the cells. The dynamic shift from the fine-tuned immune equilibrium to immune evasion during the tumor initiation, treatment and recurrence, remain unnoticed [9].

As part of routine diagnostic and prognostic assessment in clinics, the incorporation of immune phenotyping may provide significant prognostic information, which can be utilized to identify patients with a high risk of recurrence, and optimize the personalized treatment strategies accordingly. HCC develops in the background of chronic inflammation and continuous immunosuppression, so it makes sense for tumor-associated immune factors to predict recurrence in HCC [10]. Herein, we review the immunological modifications in HCC from tumor initiation to recurrence, and list the potential immunological factors associated with poor prognosis and disease recurrence. 

## 2. Immune Contexture of Liver: From Homeostasis to Carcinogenesis to Recurrence

Under homeostatic conditions, the liver acquires exquisite mechanisms of immune tolerance to prevent abrupt immune activation associated with continuous antigen exposure. A unique population of antigen-presenting cells, including Kupffer cells, liver sinusoidal endothelial cells, hepatic stellate cells, dendritic cells, and the conventional components of the innate immune system, such as natural killer cells, work in co-ordination for a finely tuned balance between immune tolerance to self-antigens and immunity to foreign pathogens [11]. Due to the continuous exposure of the liver to nutrients, blood-borne pathogens, gut-derived microbial agents and toxic waste substances, a local immunosuppressed state is maintained via the release of IL-10 by Kupffer cells and TGF-β by endothelial liver cells. Decreased surface expression of co-stimulatory molecules such as CD80 and CD86 leads to improper antigen processing by liver sinusoidal endothelial cells (LSECs) and abrogates antigen-specific immune surveillance. Interaction of PD-1 with PD-L1 expressed on hepatocytes, hepatic stellate cells, and Kupffer cells, induces T cell apoptosis, and immune tolerance [12,13]. Hepatic tolerance created by these diverse immunosuppressive cells residing in the liver can benefit harmless molecules. Furthermore, infectious agents such as viruses and malignant cells may ‘hijack’ the tolerogenic mechanisms for immune evasion and disease progression [14,15].

Etiologic factors such as viral hepatitis, alcohol consumption, metabolic syndrome, and NASH/NAFLD, trigger chronic liver injury [16]. Damage-associated molecular patterns (DAMPs) released by the injured hepatic cells are recognized by the innate immune cells, leading to liver and systemic inflammation. Kassel et al. reported that chronic inflammation associated with hepatitis viral infections, and autoimmune hepatitis, are positively correlated with the expression of immune checkpoints such as PD-1, and its ligands B1H1 and B7HC [17]. Similar studies suggest that PD-1^+^ circulating and tumor-infiltrating CD8^+^ T cells trigger the progression of liver cirrhosis to HCC [18]. Expansion and enhancement of regulatory T cell activity is also reported as the mediator of immune evasion in liver inflammatory conditions. Overexpressed CD4^+^ CD25^+^ Treg cells in HBV patients suppress the immune response to HBV antigen, and deny the HCC tumor antigen-specific adaptive immune response [19]. Therefore, during the transformation from inflammation to cancer, there is a suppression of effector immune cells such as natural killer cells, CD8^+^ T cells, cytotoxic macrophages and neutrophils. There is also overexpression of immunosuppressive cells such as pro-tumoral macrophages, regulatory T cells, regulatory B cells, immature DC, and pro-metastatic neutrophils. Chronic activation of inflammatory signaling pathways triggers the recruitment of immunosuppressive cells, and the continuous release of cytokines, growth factors, and pro-angiogenic factors. This disrupts antigen-specific immune surveillance. The inflammatory tumor microenvironment creates an imbalance between the tumoricidal effector response and tolerogenic immune response [20,21,22]. All these together induce the neoplastic transformation of hepatocytes.

As a classical archetype of inflammation-related malignancy, immune evasion occurs throughout the evolution of HCC via the expansion of immunosuppressive cells, failure of tumor antigen presentation and processing, imbalance between pro-inflammatory and anti-inflammatory cytokines, alteration of immune checkpoint pathways, and immune inhibitory receptor ligand interaction [23]. Bai et al. investigated the tumor immune microenvironment in HCC by integrated sequencing data analysis. Treg cells expressing immune checkpoints such as CTLA4, TIGH, and TNFRSF4 were found in abundance in the TIME of HCC. Altogether, NK cells and memory B cells with exhausted features were also identified [24]. Another extensive multi-omics analysis drew a paradoxical conclusion regarding the general assumption of higher CD8^+^ T cells and better survival. They characterized and categorized HCC into three immune subtypes. Cluster 1 showed an abundance of CD8^+^ T cells, cytotoxic lymphocytes, T follicular helper cells, TAM-M0, and Treg cells. There was also a decreased population of resting memory CD4 T cells, resting mast cells, monocytes, neutrophils, and stromal cells. Cluster 2 showed high infiltration of stromal cells, low infiltration of cytotoxic lymphocytes, and TAM-M0. The depleted cells in Cluster 1 and 2, i.e., resting memory CDT cells, resting mast cells, resting NK cells, resting dendritic cells, and stromal cells were enriched in Cluster 3 with fewer numbers of Treg, CD8 T cells, and T follicular helper cells. Cluster 1 exhibited the highest anti-tumor immunity, but also high immunosuppressive features. Clusters 2 and 3 tended to show suppressed and resting immune features, respectively. Interestingly, the study reported that the cluster with high CD8^+^ T cell infiltration, Cluster 1, showed poor overall survival, while Cluster 3 exhibited a better survival rate and low recurrence. This study reported some paradoxical conclusions against the general assumptions regarding the abundance of CD8^+^ T cells and better survival. Even though Cluster 1 had anti-tumor immunity with the highest CD8^+^ T cell infiltration, it resulted in poor overall survival, while Cluster 3 showed a better prognosis [25,26]. Another study identified four prognostics and immunotherapeutically-relevant subclasses. Class C1 showed a low level of all TME features. C2 was an immunogenic subtype with abundant expression of innate and adaptive immune cells. C3 was distinguished by up-regulated immunosuppressive pathways and down-regulated adaptive immunity. C4 was a mesenchymal subclass with significant immunosuppressive pathways and activated fibroblast overexpression. The study also validated that the C2 subclass was more likely to benefit from sorafenib or pembrolizumab therapy, suggesting that the tumor immune microenvironment can influence the clinical outcome of treatment [27].

The crucial role of the immune microenvironment in HCC development and progression led to the approval of the atezolizumab and bevacizumab combination as a first line therapy for advanced HCC in 2020 [28]. However, one study evaluated the immune landscape change during various stages of HCC and reported a continual immune tumor co-evolution peaking at the intermediate stage of HCC. This study identified the early or intermediate stage as a potential interventional phase to prevent HCC progression and recurrence. In the early tumor stage, curative treatment preserved liver function and improved the overall survival of patients. Nevertheless, a high recurrence rate in more than 80% of patients indicates the dismal part in the clinical management of HCC [29].

HCC recurrence can be early or late. The early recurrence phase occurs within 2 years of treatment and is mainly correlated with the tumor’s intrinsic biology such as tumor grade, local invasion and intrahepatic metastasis. Yao et al. performed a multi-institutional analysis of HCC recurrence post resection, and reported that 60% of patients developed early recurrence, and nearly 40% developed late recurrence. They suggested tumor characteristics such as tumor size > 5 cm, AFP > 400 µg/mL, and microvascular invasion as risk factors of early-stage recurrence. However, few pathological studies suggest that irrespective of the tumor size, microvascular invasion and intrahepatic metastasis can also occur in tumors of less than 2 cm, suggesting that even small tumors are recurrence prone [30,31]. Delayed recurrence occurs as de novo tumorigenesis triggered by an inflammatory–cirrhotic environment. Recently, studies have focused on correlating the immunological characteristic of tumor with recurrence, as the clinical and histopathological features are only partially reliable. Unitt et al. suggested the phenotyping of infiltrating lymphocytes to predict HCC recurrence [32]. CD4^+^, CD25^+^, and foxP3+ Treg cell accumulation was also suggested to predict prognosis and recurrence in HCC. Patients with high Treg showed poorer survival (stage I, n = 12; stage II, n = 16; stage III, n = 47; *p* < 0.001). Specifically, in the advanced stage, the survival time of the high Treg group (n = 21, average Treg frequency: 10.9% ± 1.9%) was significantly reduced compared with the low Treg group (n = 26, average Treg frequency: 6.4% ± 1.4%) [33].

As discussed above, the inherent complexity, as well as the multitude of connections between the immune and tumor microenvironments, renders the clinical management of HCC extremely difficult. In addition to the oncological factors, non-oncological factors, especially immune contexture, plays a critical role in executing tumor recurrence. The following sections explain immunological changes and potential immune factors to correlate with HCC recurrence after each curative treatment.

## 3. Recurrence Pattern after Curative Treatment in HCC

**Figure 1:**
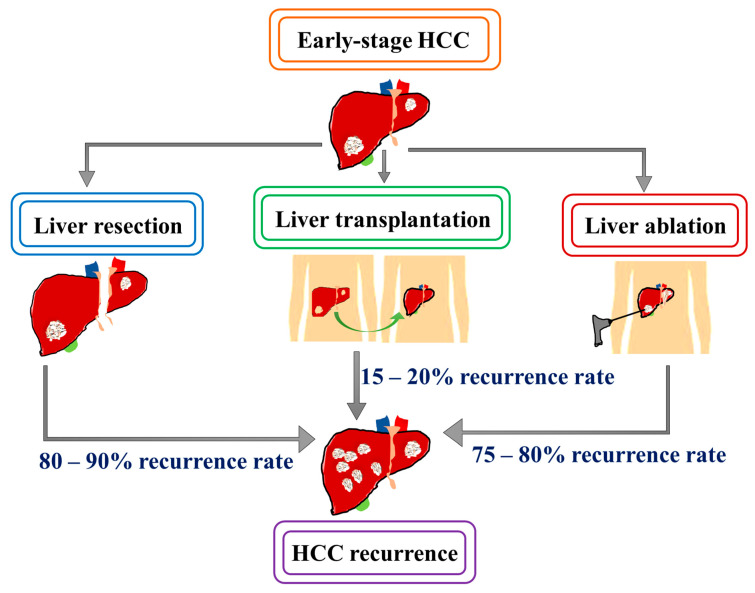
Recurrence rate after curative treatment for HCC. Despite the long-term anti-tumor response, high recurrence rates after curative treatments poses a major clinical challenge in the management of HCC.

As HCC patients represent heterogeneous features, therapeutic algorithms mostly consider the degree of liver dysfunction and patient performance status. Thus, a BCLC staging system is mostly utilized in evaluation of HCC patients. Accordingly, the early stage is characterized with single or 2–3 nodules each <3 cm with Child–Pugh score A–B and performance status 0–1 [34]. Curative interventions including surgical resection, orthotopic liver transplantation and ablative techniques are cornerstones for early-stage HCC management [6,35]. Even though curative therapies offer long term stable responses and improved survival, tumor recurrence is a crucial factor that leads to increased mortality in patients with HCC [36]. Early and late recurrence is reported in 70% of patients at 5 years after curative or locoregional therapies [37]. Identifying the patient population more susceptible to recurrence and management of recurrent HCC is essential to optimize HCC treatment strategy (Figure 1).

### 3.1. Surgical Resection

Partial hepatectomy or surgical resection mainly involves the removal of the portal territory with tumor, while protecting functional liver parenchyma. Hepatic resection and liver transplantation are the only treatments capable of curing HCC. However, recurrence within the liver remnant is reported in 80–90% of cases 5 years post-resection [38]. Studies have suggested microsatellite instability of the remnant liver, and intrahepatic metastasis from the early tumor, to trigger early recurrence within 12 months after resection, and late recurrence representing the de novo primary tumor in cirrhotic liver. Several studies correlated patient (age), tumor (vascular invasion, size stage, number of tumors, positive resection margin, alpha-fetoprotein level, mode of presentation, tumor grade), and liver (cirrhosis, hepatitis B/C status, Child–Pugh score, transaminase level, albumin level, chronic active hepatitis)-related factors with HCC recurrence [39]. In addition to these factors, tumor-infiltrating immune cell subsets significantly correlated with the HCC prognosis. Cariani et al. examined the phenotype of infiltrating cells and its prognostic relevance after liver resection. HCC infiltrate enriched with PDL1 and invariant NKT cells favored disease recurrence, while enrichment of CD4^+^ and CD8^+^ T cells was associated with better overall survival and prolonged time to recurrence [40]. High levels of memory T cells and active Th1 adaptive immunity also supported good prognosis and better survival [41].

Microvascular invasion and micrometastases are the main contributors to post-operative recurrence. The extreme vascular nature of HCC makes it a classical exemplar of poor prognostic tumor. Studies have suggested that metastasis is critically regulated by the local tissue microenvironment itself, specifically fibrotic/cirrhotic liver with significant lymphocyte infiltration. Budhu et al. reported that inflammatory features of the tumor milieu and metastatic potential of the tumor cells are the predictive factors of venous metastasis and recurrence. Microarray based gene expression profiling revealed that the transition from metastatic incline microenvironment (MIM) to metastatic averse microenvironment (MAM) was linked with the alteration of 17 relevant genes of cellular immunity and inflammatory response [42].

Ye et al. in 2003 observed a similarity in gene expression patterns between primary HCC and its corresponding metastasis. Conversely, a significant difference in gene profile was noted between metastatic-free primary HCC, and HCC with intrahepatic metastasis. Their study evidently concluded that HCC metastasis is independent of patient- and tumor-related factors [43]. In consonance with Ye et al., Budhu et al. also reported that, irrespective of the tumor size and patient age, macrophage colony stimulating factor induced Th1- to a Th2-like cytokine profile shift, promoting tumor metastasis. The suppression of pro-inflammatory Th1-like cytokines such as TNF, IFN-γ, IL-1, and IL-2, and upregulation of anti-inflammatory Th2-like cytokines including IL-4, IL-5, IL-8, and IL-10, uplift the metastatic potential of HCC cells. In this study, 17 sets of genes related to immunity and inflammation resulted in 92% accuracy and 79% sensitivity in predicting metastatic and recurrence risk, which is preferable to the conventional predictive factors of post-operative recurrence [42]. This evidence suggested the relevance of the immune signature in recurrence and pro-inflammatory based post-operative therapies to prevent HCC recurrence.

### 3.2. Orthotopic Liver Transplantation (OLT)

OLT is an effective therapy option for early-stage unresectable HCC, which offers long term stable survival. Unlike other curative approaches, OLT concomitantly resects both the tumor and underlying cirrhosis. Even though Milan criteria (single nodules < 5 cm, 2–3 nodules ≤ 3 cm) is implemented for patient selection, tumor recurrence is reported in 15–20% of cases, which suggests that morphologic criteria are not always precisely correlated with tumor biological behavior [44]. Numerous studies have identified several risk factors of disease recurrence after liver transplantation. Tumor-related factors such as tumor staging, vascular invasion, AFP level, neutrophil–lymphocyte ratio, and patient-related factors such as viral infection, hepatitis C virus (HCV) treatment, non-alcoholic fatty liver disease (NAFLD), and obesity, are frequently used in clinics to evaluate the risk of recurrence and prognosis [45,46,47]. Almost all earlier studies considered static and morphologic criteria to link with recurrence. However, recent research has complemented or combined morphologic criteria with dynamic, biological characteristics of the tumor, which has proved to be more valuable in preserving the patient outcome. The association between post-transplant immunosuppression and oncogenesis is proven in clinical settings. The inflammatory tumor microenvironment and impaired immune surveillance are essential mediators of HCC recurrence [48,49,50,51]. Studies suggest that the type of immunosuppressive agents, calcineurin inhibitors or mTOR inhibitors, and the total immunosuppressive load in the tumor microenvironment determine the risk of recurrence. Specifically, calcineurin inhibitors such as cyclosporine and tacrolimus increase the risk of recurrence, while mTOR inhibitors reduce the recurrence risk after liver transplantation [52].

Liu and team reviewed the relevance of allograft ischemia and reperfusion (IR) injury and subsequent immunological alterations in cancer recurrence after liver transplantation [53]. Hepatic IR causes allograft injury and breaks the immunological homeostasis in hepatocytes. Activated Kupffer cells and polymorphonuclear neutrophil (PMN) cells recognize DAMP secreted by the injured cells and trigger the production of cytokines, chemokines and recruitment of innate immune cells, causing inflammation [54,55]. This acute phase of inflammation within 2–24 h of reperfusion leads to increased levels of IL-6, IL-5, TNF, CXCL10, and CCL2. The altered cytokine network disturbs the dynamics between effector and regulatory T cells. IL-2, IL-10, and TGF-β mediated enrichment of Treg cells exert an immunosuppressive effect [56]. Therefore, decreased Teff/Treg ratio leads to an abrupt inflammatory response, which evolves as cancer recurrence in late phase [57,58,59]. This evidence suggests that the intra graft immune microenvironment is an important factor in predicting recurrence risk, and emphasizes the potential of immunotherapy to prevent post-transplant HCC recurrence.

### 3.3. Local Ablative Therapy (LAT)

LAT includes the destruction of tumor tissue via the thermal and chemical mode of ablation. Chemical ablation involves the use of ethanol and acetic acid, whereas thermal ablation utilizes radiofrequency, microwave, laser and ultrasound. LAT is an effective therapy for the management of early-stage unresectable HCC [60]. Even though LAT achieves a high rate of tumor ablation and a five year survival rate of 50%, the high recurrence rate of 75% represents a major concern [61,62]. Liu et al. evaluated the predictive factors of treatment outcome after percutaneous ablation in 73 HCC patients. They reported that despite 98.6% treatment effectiveness, 61 patients out of 73 (83.6%) were detected with distant recurrence during follow up. They proposed that tumor size > 2 cm promotes local tumor progression, and tumor number promotes distant recurrence after ablation [63]. Similarly, Cho et al. in 2016 evaluated the predictive factors of early massive recurrence after radiofrequency ablation in 438 patients with single small HCC. During the median follow up of 68 months, 68.9% of patients were confirmed with recurrent HCC, out of which 27 patients showed early massive recurrence [64].

In addition to the tumor-related factors, immune contexture also plays a critical role in recurrence after ablation. Even though ablation is considered as a local treatment, it exerts a prominent abscopal effect through immune modulation. Several studies have suggested that ablative therapy can enhance immunogenicity in patients. Local ablation triggers the release of tumor-associated antigens, subsequent activation of CD8^+^ T cells, and promotes systemic anti-tumor immunological effect [65]. Ali et al. in 2016 found that LAT promotes immunogenicity by activating dendritic cells and releasing endogenous adjuvants. Similarly, it was found that RFA altered the immune contexture of both innate and adaptive immune cells and exerted a tumor-specific immune response [66]. Guo et al. reported that irreversible electroporation triggered local and systemic anti-tumor immune responses by promoting the recruitment of activated T cells, especially CD8^+^ T cells, and suppressing T reg cells [67]. Xin et al. evaluated the prognostic significance of the systemic immune inflammation index after curative treatment for HCC. SII is calculated based on absolute lymphocyte, platelet and neutrophil count. Among the 509 patients enrolled in the study, the group with high SII was more susceptible to early recurrence and aggressive oncological progression. The researchers recommended SII as a comprehensive index to track immune activation before and after the therapy, being superior to other systemic inflammation markers such as platelet–lymphocyte ratio, and neutrophil–lymphocyte ratio [68]. The same immune modifications pattern was also observed in various animal models after ablative treatment [69]. The enhancement of effector immune cells such as CD8^+^ T cells, memory CD8^+^ T cells, DC, and the reduction in suppressor immune cells such as Treg cells, was reported in murine and porcine models of HCC after radiofrequency ablation [70,71,72,73].

However, Zeng et al. found that circulating PD-1/PD-L1 expression was significantly dropped post cryoablation, but eventually increased during recurrence. Multivariate analysis proposed that the circulating PD-L1 level could be a reliable marker for post-cryoablation prognosis and recurrence [74]. This evidence suggested that the anti-tumor immune response developed in response to local ablation was not strong and stable enough to prevent a recurrence. In context, an innumerable number of studies and clinical evaluations advocate combining immunotherapy with ablative treatment to sustain a favorable immune milieu to prevent recurrence.

## 4. Immunological Factors as Predictive Biomarker for HCC Recurrence

The success of HCC treatment can be determined by its ability to stabilize the disease beyond the completion of the treatment cycle, and indeed, its ability to sustain anti-tumor immune surveillance. Despite inherent immune tolerance in the liver, immune control is significantly evaded at the time of presentation of liver cancer. Treatment-induced immune alteration is also evident with curative and systemic treatments. Therefore, immune contexture comprising the components, its functional status, and density of each of the components, can significantly influence the treatment outcome, prognosis and risk of recurrence [20,75].

The HCC immune microenvironment is mainly comprised of T lymphocytes, macrophages, dendritic cells, NK cells, B and memory lymphocytes. As channeled by the vascular and lymphatic system, each component of the tumor immune infiltrate preferentially locates at unique positions and forms an organized landscape in the tumor microenvironment. T lymphocytes and macrophages reside in the tumor center and at the invasive margin, while NK cells, mast cells and neutrophils mainly present at the invasive margins. The distribution of B cell subtypes including naïve B cells and plasma cells are mostly found at the invasive margins and also in tumor lymphoid structures, bordering the tumor [76,77,78].

**Figure 2:**
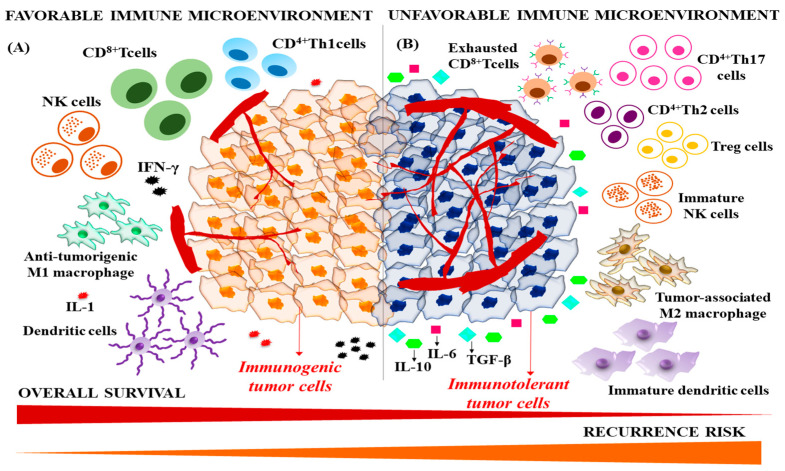
Role of immune contexture in HCC prognosis. (**A**) During the initiation of malignant transformation, effector immune cells such as CD8^+^ T cells, NK cells, Th1 CD4^+^ T cells, cytotoxic macrophages, and dendritic cells elicit a tumoricidal immune response. Therefore, an immune microenvironment with enriched effector immune cells confers effective anti-tumor immunity, good prognosis and low recurrence risk. (**B**) Tumor cells utilize various immune evasion mechanisms to escape from anti-tumor immune surveillance. Exhaustion of CD8^+^ and CD4^+^ T cells with overexpression of immune checkpoints, recruitment of regulatory T cells, polarization of macrophages to M2 phenotype, suppression of NK cells and dendritic cells leads to immune tolerance and metastatic dissemination. Therefore, an immune microenvironment with poor infiltration of effector immune cells and recruitment of immunosuppressant cells promotes poor prognosis and recurrence.

Persistent acute inflammatory signals progressively transform to tumorigenic chronic inflammation, which involves an M1 to M2 macrophage shift and release of immunosuppressive, pro-oncogenic and angiogenic factors such as VEGF, IL-6, IL-1, and TGF-β [79,80]. Upon malignant transformation, tumor-associated antigens are recognized by dendritic cells and present to MHC class I or II molecules, which leads to the priming of T cells against the tumor. Dendritic cells activate CD4^+^ T lymphocytes and CD8^+^ T lymphocytes. B cells transform into plasma cells and produce anti-tumor antibodies. Accumulation of memory B and T lymphocytes in lymphoid organs limits the metastasizing spread of cancer cells. However, tumor-microenvironment interactions hasten the evolution of immune equilibrium to immune evasion. Therefore, modification of the immune contexture plays a crucial role in prognosis and recurrence. This alludes to the potential of immunological factors to track adaptation to treatment, stability of patient response, and importantly, recurrence [78,81] (Figure 2).

### 4.1. T Lymphocyte

**Figure 3:**
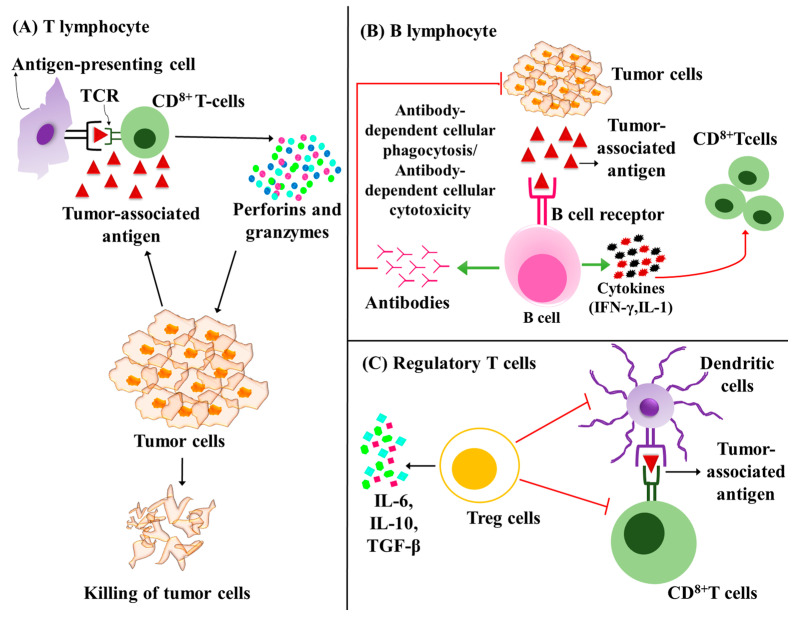
Role of lymphocytes in cancer. (**A**) T lymphocytes: CD8^+^ T cells are the major effector molecule in anti-tumor immunosurveillance. Antigen-presenting cells present MHC-bound antigen to T lymphocytes that stimulate maturation to cytotoxic CD8^+^ T cells. Activated cytotoxic T cells release cytotoxic proteins such as perforins and kill the tumor cells. (**B**) B lymphocytes: B cells can recognize tumor antigen and directly kill the cells by antibody mediated cytotoxicity or phagocytosis. B cells also function as antigen-presenting cells and the cytokines released from B cells can activate CD8^+^ T cells. (**C**) Regulatory T cells: T reg cells are suppressor type T cells which inhibit CD8^+^ T cells and dendritic cells. T reg cells also produce inhibitory cytokines such as IL-, IL-10, and TGF-β (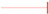 denotes inhibition).

T and B lymphocytes constitute the major effector cells of adaptive immunity. In addition to circulating T cells, the liver harbors an abundant population of resident lymphocytes which include CD8^+^ T cells, NK cells and NK T cells. T cell receptor (TCR) expressed on the surface of T cells recognizes the tumor antigen by interaction with the major histocompatibility complex (MHC) molecule in antigen-presenting cells. In order to stabilize the interaction of TCR and antigen-bound MHC, co-receptors are required. Helper T cells express CD4 co-receptor, and cytotoxic T cells express CD8 co-receptor. CD3 co-receptor is essential in the activation of CD8^+^ and CD4^+^ T lymphocytes. CD8^+^ T cells lyse the cancer cells by releasing granzyme B, IFN-γ, perforins, and TNF-α (Figure 3A) [82].

Fridman et al. analyzed the prognosis of patients in relation to the nature of immune cells. In light of more than 100 published articles, they summarized the effect of T cells on the clinical outcome, and found that a strong infiltration of CD4^+^ T cells, CD8^+^ T cells and CD45RO+ memory T cells were positively correlated with prolonged recurrence-free survival following surgical resection in HCC patients [83]. Similarly, a high CD4^+^/CD8^+^ T cell ratio prevented recurrence after liver transplantation. Some studies suggest that in addition to the density, the quality or functional status of T cells also influences its anti-tumor efficacy [32,84]. Gabrielson et al. suggested that PD-L1 expression and immune score based on CD4^+^ and CD8^+^ T cells density can be validated as a prognostic marker in HCC after surgical resection. Irrespective of the high density of T lymphocytes in tumor infiltrate, T cell exhaustion controlled by elevated expression of B7-H1, PD-1, CTLA4, CD160, LAG-3, Tim-3+ and T cell aging mediated by upregulation of CD57, KLRG-1, and Tim-3 was found to play a crucial role in triggering HCC recurrence after curative treatments [85].

As mentioned earlier, CD4^+^ T cells are helper T cells, which assist B-cell-mediated antibody production, phagocytic activity of macrophages, and immune cell recruitment to the infected area. CD4 T cells mainly differentiate into three types with differential cytokine production and function: Th1, Th2 and Th17. Th1 cells support cellular immunity and produce IL-2 and IFN-γ. Th2 cells mainly act on B cells and produce IL-4, IL-5 and IL-13. Th17 cells act on fibroblast, endothelial and epithelial cells and produce IL-17, IL-21, and IL-22. In general, Th1 cells are associated with better anti-tumor immune surveillance, while Th2 and Th17 cells trigger pro-tumorigenic inflammation and mediate relapse [86,87]. Song et al. found that patients with recurrent HCC expressed low frequencies of IFN-γ producing Th1 and Tc1 cells, and high frequencies of Foxp3+ Treg cell in resected tumor as well as in peripheral blood [88]. A comparative study of immunogenomics characteristics of conventional HCC and HCC with immune stroma (IS) revealed that HCC IS subtype was associated with better prognosis and recurrence-free survival with elevated infiltration of CD8 T cells. Together with this, high levels of EBV+ (Epstein–Barr virus) tumor-infiltrating lymphocytes were associated with poorer recurrence-free (aHR 25.48; *p* < 0.001) and overall (aHR 9.6; *p* = 0.003) survival. Immunohistochemical, immune or cytofluorometric techniques are mainly used to evaluate tumor-infiltrating lymphocytes with targeted gene expression and circulating lymphocytes [89].

### 4.2. B Lymphocytes

B cells play the major role of the adaptive humoral immune system and are responsible for antigen-specific antibody production (Figure 3B). Similar to T lymphocyte infiltration, tumor-infiltrating B cells (TIB) also contribute to a positive prognosis in HCC [90]. Shi et al. suggested margin infiltration of CD20+ B cells as a protective factor of HCC [91]. Among the five subtypes of tumor-infiltrating B lymphocytes, a high level of naïve B cells and CD27-switched memory B cells were linked with high IFN-γ secretion and, therefore, are a good prognosis marker of HCC. Prolonged overall survival was observed with high infiltration of CD20+ (63 versus 41 months), Bn (63 versus 43 months), Ig M+ Bm (63 versus 43 months), and CD27-Sw Bm (63 versus 43 months) subsets of B cell [92]. In addition to the direct anti-tumor effect by secreting antibodies against tumor-associated antigens, TIB cells increase CD8^+^ T cell infiltration mainly by the IFN-γ mediated pathway. IL-2 and IFN-γ secreted by TIB can also activate NK cells. As for Treg cells, regulatory B cells mediate poor prognosis and fast recurrence by suppressing effector immune cells such as CD4^+^ and CD8^+^ T cells, and NK cells [93,94].

### 4.3. Regulatory T Cells

Treg cells are a subtype of CD4^+^ T cells that suppress other immune cells and immune surveillance (Figure 3C). Natural Treg cells are the predominant Treg cell type, which are characterized by positive expression of CD4, CD25 and fox head box P3 (fox p3). Induced T reg cells develop from CD4^+^ and fox P3-T cells. Treg cells induce oncogenic inflammation by fas/fasL pathway, eliminate effector immune cells such as NK cells and CD8^+^ T cells by granzyme/perforin mediated pathway, and secrete suppressive soluble messengers such as TGF-β, IL-10 and adenosine [95]. 

Even though both Treg cell types suppress the immune response, foxp3 expression regulates its effect on the clinical outcome of HCC patients [96]. Wang et al. evaluated foxP3 expression and clinical characteristics of HCC. They found that approximately 50% of tumors were confirmed with positive foxP3 expression, with no expression in normal liver tissue [97]. Similarly, Huang et al. performed a meta-analysis evaluating the relevance of foxP3 plus T cells on GI cancers and demonstrated that a foxP3 plus T cell population is associated with reduced survival and increased recurrence in HCC patients [98]. 

The Treg cell population showed a progressive elevation from early to advanced stage, suggesting its critical role in HCC progression. Even though curative treatment exerted a positive regulatory role on anti-tumor immune surveillance by suppressing Treg cells, it gradually reverted back to elevated Treg mediated immunosuppression which hastened disease recurrence. Two independent studies conducted by Fu et al. and Gao et al. confirmed that elevated Treg cells lead to CD8^+^ T cell inhibition and poor prognosis in HCC. Patients with high intratumoral Treg and low activated cytotoxic T lymphocytes (CTL) exhibited five year overall survival (OS) and disease-free survival (DFS) of 24.1% and 19.5%, respectively, compared with 64% OS and 59.4% DFS for patients with low Treg and high CTL [33,99]. Li et al. suggested that CXC10/CXCR3 signaling-mediated mobilization of Treg cells in liver promotes HCC recurrence after liver transplantation. Cumulative recurrence was higher in HCC recipients with small-for-size liver graft after transplantation (19.3%). Among them, the patients with recurrence showed more circulating Treg cells at the first, third, and sixth month after transplantation [96,100]. All this evidence proposes Treg cell expression in HCC tumor and its microenvironment as a biomarker to predict HCC recurrence after curative treatment.

### 4.4. Macrophages

**Figure 4:**
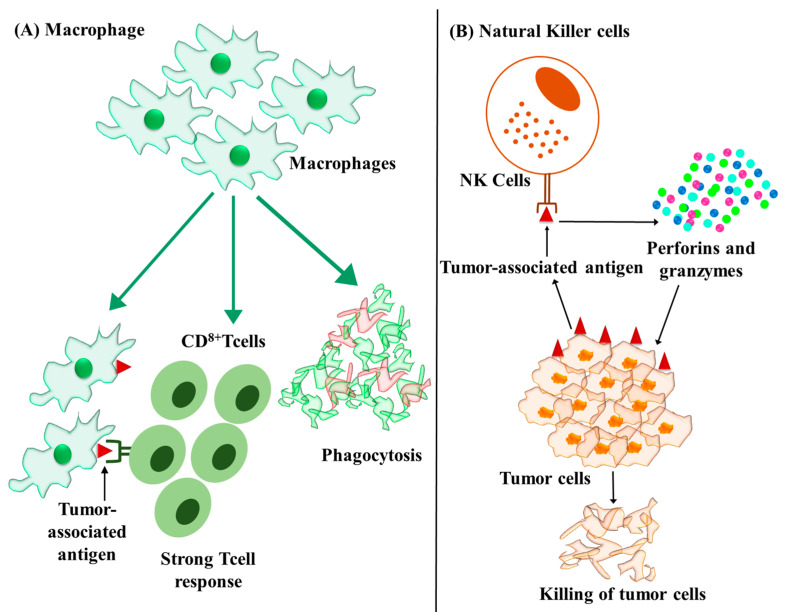
Role of macrophage and natural killer cells in cancer. (**A**) Macrophages are phagocytic cells, which function as antigen-presenting cells and an inflammatory modulator via release of various cytokines. (**B**) Natural killer cells (NK cells) can directly kill tumor cells by the release of cytotoxic proteins such as perforins and granzymes without any priming.

Liver macrophages comprised of Kupffer cells and monocyte-derived macrophages represent the most important cellular component of liver involved in homeostasis as well as in hepatocarcinogenesis. In addition to phagocytosis, macrophages are also involved in antigen presentation, inflammation and immune modulation by the release of various cytokines (Figure 4A) [101,102]. Macrophages can be differentiated into two polarization phenotypes: M1 and M2. Classically activated M1 macrophages are pro-inflammatory in nature by releasing cytokines such as IL-1, IL-12, IL-6, IL-23, TNF-α, and CXCL-5, and also promote antigen presentation and T cell functionality. Alternately activated M2 macrophages are anti-inflammatory in nature with high level anti-inflammatory cytokines including IL-10 [103,104,105,106,107,108].

Dense macrophage infiltration is dominant in tumor cells, where it is termed as tumor-associated macrophages (TAM). TAM are more relevant in the context of HCC as they represent the prepotent leukocyte population in HCC and involve HCC progression and recurrence [109]. Generally, TAM accumulate in the tumor stroma and are also found in the tumor center/invasive margins. Chronic inflammatory milieu in HCC is balanced by anti-inflammatory M2 macrophages and pro-inflammatory M1 macrophages. However, as the tumor progresses, the polarization phenotype is mostly shifted towards M2 which suppresses adaptive immunity and promotes HCC proliferation and metastasis [110,111,112].

Proteomics and transcriptomic analysis using a TCGA data set showed that HCC mostly harbors TAM, Treg and resident NK cells, which lead to poor prognosis after surgical resection [113,114]. Dong et al. reported that M2 macrophages promote tumor growth and invasiveness in HCC. Similarly, Dong et al. evaluated TAM as a predictive marker for HCC prognosis and found the increased level of CD206+ M2 macrophages and decreased level of CD80+ M1 macrophages promoted HCC to an aggressive phenotype with poor overall survival (*p* = 0.027 and *p* = 0.024, respectively) and increased time to recurrence (*p* = 0.037 and *p* = 0.031, respectively). Importantly, a combined estimation of CD206 and CD80 showed better indication of overall survival (*p* = 0.017) and time to recurrence (*p* = 0.024) than individual estimation [115].

Apart from the polarized macrophages, resting macrophages (M0) also are important in HCC progression. Zhang et al. recently analyzed the infiltration of M0 macrophages and related gene expression in HCC to construct a risk score model. They revealed prominent M0 macrophage infiltration in HCC tissue as compared with normal tissue, which is linked with HCC progression, poor overall survival and early recurrence. The univariate Cox analysis model showed that the proposed risk score model was negatively correlated with overall survival, and patients in the high risk group were associated with poor prognosis in terms of progression-free interval, disease-free interval, and disease-associated survival [116]. According to Kuang et al., enrichment of CD68+ TAM in HCC induces Th17 cell expansion and promotes HCC progression. Similarly, intratumoral macrophage-mediated Treg cell activation was also reported in many studies, suggesting the potential role of TAM to follow treatment response and predict risk of recurrence [117].

### 4.5. Natural Killer Cells

Natural killer cells belong to the lymphocyte class of immune cells. Hepatic NK cells contain liver resident or transient conventional NK cells, which account for 50% of the hepatic lymphocyte population and have significant involvement in hepatocarcinogenesis [118]. The natural killing effect is important to prevent/control the malignant transformation of hepatic cells. In contrast with cytotoxic T lymphocytes, NK cells do not require priming and can directly kill the tumor cells (Figure 4B). Cytokines released from NK cells such as IFN-γ and TNF-α can indirectly enhance the immune response by macrophages and dendritic cells [119].

An inflammatory tumor microenvironment enriched with proliferating immune cells such as T cells and CD56+ NK cells improves overall survival in HCC patients [120]. Lee et al. evaluated NK cell activity and its association with stage and recurrence pattern in HCC, to construct a risk score model for patients undergoing RFA/surgical resection. They found that NK cells at the time of diagnosis was an important parameter to consider for staging. An IFN-γ producing NK cell population showed progressive elevation from BCLC 0 to D stage. However, one month after the curative treatment, patients with <45% IFN-γ producing NK cells were at high risk of recurrence [121]. Taketomi et al. also reported the same pattern, concluding that <30% pre-operative NK cell activity was associated with limited response to treatment, poor disease-free survival, and increased relapse. Cytotoxic function of NK cells as analyzed by a high level of cytotoxic granules, CD3ζ and low level of natural cytotoxic receptor-NKG2A (inhibitory protein) co-expression, and IFN-γ producing nature, were the main characteristics of NK cells associated with better overall survival and recurrence-free survival [122].

Licensing is essential for the functional competency of NK cells which involves interaction between killer immunoglobulin-like receptor (KIR) and human leukocyte antigen (HLA). KIR genes and its corresponding ligands exhibit diverse polymorphism and exert a wide range of immune responses to HCC cells. One study demonstrated that KIR-HLA genotypes can predict the risk of recurrence after hepatectomy. They found that, among the five functional compound genotypes of NK cells such as KIR2DL1-C2, KIR2DL2-C1, LIR3DLK-BWG, and KIR3DL2-A3/11, the presence of any single KIR-HLA gene did not show any correlation with recurrence. Interestingly, when there were multiple genotypes of KIR-HLA, more than two genes increased the risk of recurrence [123].

Invariant NK cells or classical NKT cells are T cells expressing an invariant T cell receptor, but share the functional characteristics of NK cells. Even though iNKT is comparatively rare in human blood, its ability for rapid-enhanced secretion of various cytokines makes it an important immune regulatory cell. Xiao et al. investigated the prognostic value of intratumoral iNKT cells and IFN-γ in HCC after surgical resection. Patients with low levels of iNKT cells and IFN-γ exhibited an aggressive phenotype and the worst prognosis. Patients with a low level of iNKT cells and IFN-γ showed a hazard ratio of 2.784 for overall survival and 2.673 for recurrence-free survival [124].

In addition to the association between immune cell density and risk of recurrence, many proteomic and transcriptomic studies have proposed other immune markers of HCC. Xing et al. summarized the utility of autoantibodies against tumor-associated antigens (TAA) as an immunodiagnostic marker of HCC. In this study, they designed a microarray based on important TAA and found that a 14 TAA panel showed superior clinical value for HCC diagnosis with 69% sensitivity and 83% specificity. Interestingly, approximately 50% of patients diagnosed with HCC by this autoantibody-based microarray had normal AFP levels, which suggested the superior predictive value of immunological factors in HCC [125]. Wang et al. constructed an HCC recurrence model based on immune-related long non-coding RNA (lnRNA), as lnRNAs are important regulators of gene expression and immune response. They identified nine lnRNAs closely related to disease-free survival which could be validated as potential biomarkers to predict HCC recurrence [126]. Similarly, Ye et al. compared gene expression data of HCC patients and normal tissue samples and selected differentially expressed immune related genes in HCC samples, and constructed an immune-related gene signature based on eight genes such as CHGA, RAETIE, FGF9, GIP, NROB1, IL20RA, ESRRG, and GNNRH2 to predict the HCC prognosis [127]. Another research team designed 15 immune-related gene signatures as a prognostic model for an HCC-based TGCA dataset using Cox regression analysis [128]. Similarly, a recent study used an immunohistochemistry classifier assay and constructed a nine factor-based HCC-IHC classifier to predict early recurrence after surgical resection. They validated the model by comparing the 5 year relapse-free survival rate of the low HCC-HCC classifier (46%) vs. the high HCC-IHC classifier (26.7%) and presented this model as a superior system with better accuracy than the conventional staging systems [129].

## 5. Immunotherapeutic Approaches to Prevent HCC Recurrence

Over the past few years, evasion of the host anti-tumor immune surveillance has been recognized as the important hallmark of carcinogenesis. This resulted in the development of immunotherapy as the fifth pillar of cancer management alongside surgery, radiotherapy, chemotherapy, and targeted therapy [130]. In addition to curative or systemic treatment, a favorable immune microenvironment is the critical factor responsible for eliminating tumor cells from the body and maintaining stable immunosurveillance to prevent recurrence [131]. Multiple studies have proposed immune monitoring to predict disease prognosis and recurrence, and proved superior efficacy of immune contexture phenotyping over the conventional oncological markers of tumor progression.

Tyrosine kinase inhibitors (TKIs) including sorafenib and lenvatinib were the first- line therapy for advanced HCC for the last ten years before the approval of the combination of anti-PDL1 antibody;atezolizumab with VEGF inhibitor;evacizumab in 2020 [132]. Immune checkpoint inhibitors (ICIs) showed superior efficacy in terms of overall survival and progression free survival than TKIs. Even though drug therapy is preferred for advanced HCC, de novo tumorigenesis and high recurrence rate after curative treatment urged the implementation of neo-adjuvant or adjuvant drug therapy to prevent relapse. However, TKIs failed to exhibit survival benefits in clinical trials as an adjuvant therapy with curative treatment [133]. The impact of the immune signature in HCC recurrence and excellent results of ICIs in advanced HCC supported the combination of immunotherapy with curative treatment to manage early recurrence. Importantly, neo-adjuvant or adjuvant immunotherapy is also under clinical evaluation for other cancers such as triple negative breast cancer, Merkel cell carcinoma, melanoma, and colon cancer [134,135,136].

In addition to role of immunological factors to predict recurrence risk, a combination of immunotherapeutic approaches with curative or systemic treatment can enrich effector immune molecules and surpass immunosuppressive signals [137,138]. Importantly, the results of a recently published single arm phase I b study suggested the potential of pre-operative therapy to convert unresectable HCC to resectable with enhanced anti-tumor immunity. This study enrolled 15 patients ineligible for resection due to high-risk features, who underwent two week cabozantinib treatment followed by four cycles of anti-PD L1 antibody, nivolumab. After 10 weeks, 12 patients underwent successful resection and 42% of patients exhibited major pathologic responses with enrichment of effector T cell functionality [139]. Many ongoing clinical trials are investigating immune checkpoint inhibition after surgery or ablative therapy for HCC in order to prolong recurrence-free survival (Table 1). A phase II trial of peri-operative camrelizumab–apatinib combination exhibited significant efficacy in patients with resectable HCC in terms of 1-year recurrence-free survival rate (54.5%), major pathological reactions (18%), and complete pathological response (6%). Importantly, the combination resulted in manageable toxicities in all patients [140]. The CheckMate 9DX study with nivolumab, and the Keynote 937 study with pembrolizumab, are under phase 3 trials, investigating recurrence-free survival in HCC patients who have undergone surgical resection or ablation [141]. However, the recently published final result of the NIVOLVE (adjuvant nivolumab for HCC after surgical resection or radiofrequency ablation) trial showed that the adjuvant Nivolumab after resection or RFA in HCC resulted in a 1-year recurrence-free survival rate of 78.6% and recurrence-free survival of 26.3 months [142]. Even though this trial of ICI monotherapy was more effective than the STORM trial (sorafenib as adjuvant treatment following resection or ablation in the prevention of recurrence of hepatocellular carcinoma), the NIVOLVE trial was less effective in preventing recurrence in patients with high Treg infiltration or beta-catenin activation. Therefore, recent ongoing phase III trials such as EMERALD-2 and IMbrave050 combined ICI and anti-VEGF antibody as adjunct therapy after curative treatment, and may result in improved overall and recurrence-free survival [143].

Despite the significant improvement in overall survival and recurrence-free survival, immune checkpoint inhibition can produce a spectrum of immune-mediated adverse events (IMAE) affecting any of the organs, which is distinguishable from chemotherapy-related adverse events. The pattern of adverse events depends on the agent, its dose, duration of treatment, and patient-related factors [144]. In the CheckMate 040 trial, 50% of patients with a high dose of ipilimumab and 24% of patients with a low dose of ipilimumab were treated with corticosteroids to manage IMAE [145]. Importantly, the combination of ICI with TKI showed more severe toxicity than ICI monotherapy. The combination of pembrolizumab and lenvatinib reported some lethal adverse events in clinical trials [146]. In contrast, the atezolizumab and bevacizumab combination was better tolerated in patients. Hypertension was the only frequently reported adverse drug reaction. However, bevacizumab is associated with gastrointestinal bleeding, with high risk in cirrhotic patients. Another challenge is the timing of neo-adjuvant/adjuvant therapy. Risk of IMAEs is high with a long cycle of treatment. Liu et al. suggested a short duration between the first administration of ICI and surgery, ideally 4–5 days to provide optimum efficacy [147]. A predictive biomarker can be utilized to evaluate the response to immune checkpoint inhibition. Target molecule expression such as PD-1 and PDL1, neo-antigens, immune gene signature, T cell clonality, tumor-infiltrating lymphocytes, and circulating lymphocytes can be estimated to monitor ICI therapy as well as to design personalized therapy regimens [148]. Together with this, the lack of a validated endpoint to monitor the response of adjuvant ICI is another limitation. Oncology trials focus on survival benefits as the endpoint rather than a complete cure. Different clinical trials use different endpoints which may lead to a biased estimation of tumor response. In the nivolumab/ipilimumab trial, the pathologic response rate was found to be 78%, while the overall response rate was only 23% [133].

**Table 1:** Clinical trials combining immunotherapy with curative treatment for HCC.

Trial Name/NCT Identifier	Curative Treatment	Neo-Adjuvant or Adjuvant Immunotherapy	Intervention Details	Primary Endpoints	Number of Participants	Phase and Status
PRIMER-1 NCT05185739 Intervention model: parallel assignment. Masking: open label.	Surgical resection.	Pembrolizumab (200 mg IV every 3 weeksfor two cycles) levantinib combination (8 or 12 mg PO once daily for 6 weeks).	Six-week pre-operative therapy of pembrolizumab–levantinib combination followed by up to 12 months’ treatment with permbrolizumab after resection.	Combination results in less than 10% viable cells at the time of resection. Relapse-free survival at 12 months from surgery.	60	Phase 2. Recruiting.
DYNAMIC/NCT04954339 Intervention model: single group assignment. Masking: open label.	Surgical resection.	Atezolizumab (1200 mg) plus bevacizumab (15 mg/kg).	Two cycles of pre-operative therapy of atezolizumab plus bevacizumab followed by four cycles of combination after surgery.	Rate of complete pathological response (absence of viable tumor cells in any nodule). Dynamic changes in the immune infiltrate following treatment. Recurrence-free survival.	45	Phase 2. Recruiting.
NCT03510871 Intervention model: single group assignment. Masking: open label.	Surgical resection.	Nivolumab plus ipilimumab.	Nivolumab 3 mg/kg plus ipilimumab 1 mg/kg intravenously on day 1 of each cycle (every 3 weeks). Eligible patients undergo surgery.	Percentage of patients with tumor shrinkage. Safety of nivolumab plus ipilimumab as adjuvant therapy.	40	Phase 2. Active. As of February 2021, the progression-free survival was 13.4 months [149].
NCT03867370 Intervention model: sequential assignment. Masking: open label.	Surgical resection.	Arm A: Toripalimab (480 mg i.v single dose). Arm B: Toripalimab (480 mg i.v single dose) plus lenvatinib (12 or 8 mg daily orallybased on body weight).	Single dose pre-operative toripalimab, and after surgery, toripalimab for 48 weeks.	Pathological response rate.	40	Phase Ib/II Active. To September 2021, out of 18 enrolled patients, 16 were evaluable. Three patients achieved major pathologic response (MPR, residual tumor in <50% tumor bed) [150].
MEDI4736 NCT05194293 Intervention model: single group assignment. Masking: open label.	Surgical resection.	Durvalumab–regorafenib.	Durvalumab–regorafenib combination every 28 days until surgery or up to 2 years post registration unless there is unacceptable toxicity.	Objective response rate, defined as a complete response or partial response. Recurrence-free survival.	27	Phase 2. Not yet recruiting.
NCT04224480 Intervention model: single group assignment. Masking: open label	Surgical resection.	Pembrolizumab 200 mg as intravenous infusion every 3 weeks.	Single dose of pembrolizumab prior to surgery. Adjuvant treatment with pembrolizumab will be administered 4 weeks after the surgery.	Number of subjects with recurrence. Number of CD8^+^ and Ki67+ T cells found in resected tumor from subjects.	45	Recruiting Phase 1.
NIVOLVE UMIN 00002664 Intervention model: single group assignment Masking: open label.	Resection or ablation.	Nivolumab.	Nivolumab (240 mg/body) every 2 weeks (eight cycles), followed by nivolumab (480 mg/body) every 4 weeks (eight cycles) within 6 weeks after SR or RFA.	One year recurrence-free survival rate of 78.6% and recurrence-free survival of 26.3 months [135].	55	Completed.
CheckMate 9DX NCT03383458 Allocation: randomized. Intervention: parallel assignment. Masking: quadruple (participants, care providers, investigators and outcomes assessors).	Resection or ablation.	Arm A: Nivolumab. Arm B: Placebo comparator.	Nivolumab. Specified dose on specified days after resection or ablation.	Recurrence free survival.	545	Phase 3. Ongoing.
KEYNOTE-937 NCT03867084 Allocation: randomized. Intervention: parallel assignment. Masking: double (participants, investigators).	Resection or ablation.	Arm A: Pembrolizumab. Arm B: Placebo comparator (IV infusion of 0.9% NS).	Intravenous pembrolizumab at 200 mg on day 1 of each 21-day cycle for up to 17 cycles.	Recurrence-free survival. Overall survival.	950	Phase 3. Active.
EMERALD-2 NCT03847428 Allocation: randomized. Intervention: parallel assignment. Masking: quadruple (participants, care providers, investigators and outcomes assessors).	Hepatic resection or ablation.	Arm A: Durvalumab 1120 mg (Q3W) plus bevacizumab 15 mg/kg (Q3W). Arm B: Durvalumab 1120 mg (Q3W) plus bevacizumab placebo (Q3W). Arm C: Durvalumab placebo (Q3W) plus bevacizumab placebo (Q3W).	Durvalumab in combination with bevacizumab in high risk of recurrence HCC patients after curative treatment.	Recurrence-free survival.	908	Phase 3. Active.
IMbrave 050 NCT04102098 Allocation: randomized. Intervention: parallel assignment. Masking: open label.	Hepatic resection or ablation.	Arm A: Atezolizumab (1200 mg IV infusion on day 1 of each 21-day cycle) plus bevacizumab (IV infusion at a dose of 15 mg/kg on day 1 of each 21-day cycle). Arm B: Active surveillance comparator. Active surveillance of participants.	Participants will receive atezolizumab plus bevacizumab until unacceptable toxicity after resection or ablation.	Recurrence-free survival.	668	Phase 3. Active.

## 6. Conclusions

HCC is the second leading cause of cancer-related mortality, and global incidence is expected to rise 55% by 2040. Cure of HCC is only possible at the early stage of the disease by curative interventions. Even though curative treatments provide a long-term response, the high rate of recurrence is limiting its potential utility. Alongside the anatomical features of the tumor, the immune microenvironment signature as defined by its components, density, functionality, organization within the tumor, and expression pattern of immune related genes, has a critical role in disease prognosis. Recently, many researchers have evaluated alterations in the immune equilibrium, such as suppression of cytotoxic T lymphocyte population, enrichment of exhausted T cells, Treg cells, and tumor-associated macrophages from the premalignant stage to recurrence, and suggested tumor-associated immune factors as marker candidates to predict the risk of recurrence in HCC. This warrants extensive research to identify the relevant immune contexture of patients in controlling treatment response, to validate its potential to predict recurrence risk and to design personalized therapeutic regimens. With the advancement of technology, a range of methods such as immunohistochemistry, multiplex immunofluorescence, genomic or transcriptomic analysis, mass spectrometry, or even blood-based immunological parameters are available, which can be effectively utilized, characterizing the immune contexture to identify the patients at high risk of recurrence. In spite of recent trends towards immunotherapy, only a fraction of patients are responsive, also causing serious life-threatening adverse events. An immune class of HCC characterized by significant immune infiltration, specifically T lymphocyte infiltration with overexpression of PD-1/PD-L1 and CTLA4, is best suited for immune checkpoint inhibitor therapy. Patients with poor T lymphocyte infiltration showed high recurrence and poor response to immunotherapy with ICI. Correct patient selection is the key factor to the success of adjuvant immunotherapy. Therefore, immunophenotyping can be employed to evaluate the immune signature, patient selection, and to monitor treatment response. The ongoing clinical trials combining immunotherapy with curative treatment are paving a new era of HCC management, which offer stable anti-tumor response, strong anti-tumor immune surveillance, and recurrence-free survival.
